# Innova4Health: an integrated approach for prevention of recurrence and personalized treatment of Major Depressive Disorder

**DOI:** 10.3389/frai.2024.1366055

**Published:** 2024-05-07

**Authors:** Francesco Monaco, Annarita Vignapiano, Martina Piacente, Federica Farina, Claudio Pagano, Alessandra Marenna, Stefano Leo, Corrado Vecchi, Carlo Mancuso, Vincenzo Prisco, Davide Iodice, Annarosaria Auricchio, Roberto Cavaliere, Amelia D'Agosto, Michele Fornaro, Marco Solmi, Giulio Corrivetti, Alessio Fasano

**Affiliations:** ^1^Department of Mental Health, ASL Salerno, Salerno, Italy; ^2^European Biomedical Research Institute of Salerno (EBRIS), Salerno, Italy; ^3^Innovation Technology e Sviluppo (I.T.Svil), Salerno, Italy; ^4^Ufficio Trasferimento Tecnologico, Università degli Studi di Cassino e del Lazio Meridionale, Cassino, Italy; ^5^Istituto Polidiagnostico D'Agosto & Marino, Nocera Inferiore, Italy; ^6^Department of Neuroscience, Reproductive Sciences, and Odontostomatology, Clinical Section of Psychiatry and Psychology, University School of Medicine Federico II, Naples, Italy; ^7^Department of Psychiatry, University of Ottawa, Ottawa, ON, Canada; ^8^Department of Mental Health, The Ottawa Hospital, On Track: The Champlain First Episode Psychosis Program, Ottawa, ON, Canada; ^9^Clinical Epidemiology Program, Ottawa Hospital Research Institute, University of Ottawa, Ottawa, ON, Canada; ^10^School of Epidemiology and Public Health, Faculty of Medicine, University of Ottawa, Ottawa, ON, Canada; ^11^Department of Child and Adolescent Psychiatry, Charité—Universitätsmedizin, Berlin, Germany; ^12^Department of Pediatrics, Massachusetts General Hospital for Children, Harvard Medical School, Division of Pediatric Gastroenterology and Nutrition, Boston, MA, United States; ^13^Mucosal Immunology and Biology Research Center, Massachusetts General Hospital for Children, Boston, MA, United States

**Keywords:** Major Depressive Disorder, air pollution, digital biomarkers, wearable technology, artificial intelligence

## Abstract

**Background:**

Major Depressive Disorder (MDD) is a prevalent mental health condition characterized by persistent low mood, cognitive and physical symptoms, anhedonia (loss of interest in activities), and suicidal ideation. The World Health Organization (WHO) predicts depression will become the leading cause of disability by 2030. While biological markers remain essential for understanding MDD's pathophysiology, recent advancements in social signal processing and environmental monitoring hold promise. Wearable technologies, including smartwatches and air purifiers with environmental sensors, can generate valuable digital biomarkers for depression assessment in real-world settings. Integrating these with existing physical, psychopathological, and other indices (autoimmune, inflammatory, neuroradiological) has the potential to improve MDD recurrence prevention strategies.

**Methods:**

This prospective, randomized, interventional, and non-pharmacological integrated study aims to evaluate digital and environmental biomarkers in adolescents and young adults diagnosed with MDD who are currently taking medication. The study implements a sensor-integrated platform built around an open-source “Pothos” air purifier system. This platform is designed for scalability and integration with third-party devices. It accomplishes this through software interfaces, a dedicated app, sensor signal pre-processing, and an embedded deep learning AI system. The study will enroll two experimental groups (10 adolescents and 30 young adults each). Within each group, participants will be randomly allocated to Group A or Group B. Only Group B will receive the technological equipment (Pothos system and smartwatch) for collecting digital biomarkers. Blood and saliva samples will be collected at baseline (T0) and endpoint (T1) to assess inflammatory markers and cortisol levels.

**Results:**

Following initial age-based stratification, the sample will undergo detailed classification at the 6-month follow-up based on remission status. Digital and environmental biomarker data will be analyzed to explore intricate relationships between these markers, depression symptoms, disease progression, and early signs of illness.

**Conclusion:**

This study seeks to validate an AI tool for enhancing early MDD clinical management, implement an AI solution for continuous data processing, and establish an AI infrastructure for managing healthcare Big Data. Integrating innovative psychophysical assessment tools into clinical practice holds significant promise for improving diagnostic accuracy and developing more specific digital devices for comprehensive mental health evaluation.

## 1 Background

### 1.1 Epidemiology of Major Depressive Disorders

Close to one billion individuals globally grapple with mental health conditions, Major Depressive Disorders (MDD) is one of the most widespread among them (Institute of Health Metrics and Evaluation, 2022). MDDs are primarily distinguished by a reduction in energy and interest in daily activities, feelings of sadness, impaired concentration, sleep disturbances, emptiness, irritability, and the presence of suicidal ideation, planning, and attempts. With over 700,000 suicides reported annually, MDD stands as the fourth leading cause of death among individuals aged 15–29 years old (Malhi et al., 2018; World Health Organization, 2022). The onset of the disease occurs during adolescence in nearly 50% of cases, and a high risk of recurrence and chronicity across the lifespan in 5% of cases. Depression is about 50% more common among women than men (Lam et al., 2016).

After experiencing a first depressive episode, the recurrence rate within 3 years is more than 30%, and some patients have recurrent episodes; thus, the course of the disease can be chronic, placing a heavy burden on both patients and society (Kuehner, 2017). Despite its high prevalence, depression remains undiagnosed and untreated in half of all cases (Kohn et al., 2004).

### 1.2 Biomarkers of MDD

The causes of MDD are complex and include multiple converging factors, such as genetic, immune system, endocrine factors, and stress-related psychosocial conditions (Ruiz et al., 2022). The specific pathways contributing to MDD onset and progression remain unclear (McIntyre et al., 2014; Lai et al., 2019; Wölfer et al., 2019), partly due to its clinical heterogeneity. MDD might have biomarkers from various sources, including inflammation, immune responses, neurotrophins, neurotransmitters, metabolic processes, and neuroendocrine systems (Belzeaux et al., 2018; Mora et al., 2018). Consequently, the use of any potential objective biomarker for accurately diagnosing depression warrants in-depth investigation.

The inflammatory immune-mediated hypothesis supported that immune dysfunction may contribute to both comorbid depression (associated with clinical inflammation) and primary depression in some individuals with low-grade inflammation indices (Averna et al., 2020). Literature data show that inflammatory biomarkers are altered in individuals with MDD (Soskin et al., 2012; Beurel et al., 2020; Osimo et al., 2020). The literature revealed significantly elevated levels of various inflammatory biomarkers, including CRP, IL-3, IL-6, IL-12, IL-18, sIL-2R, and TNFα, in individuals with MDD. Additionally, Osimo et al., observed a decreased mean-scaled variability (CVR) in patients with MDD for CRP, IL-12, and sIL-2R, indicating a more consistent inflammatory phenotype. The overall findings support the association between MDD and a pro-inflammatory state, underscoring the importance of understanding inflammation's role in shaping the clinical phenotype and affecting treatment response (Soskin et al., 2012; Beurel et al., 2020; Osimo et al., 2020).

Several studies support an important correlation between air pollution and depression. Exposure to air pollution has been found to lead to depression-like behaviors in animal studies, and exposure to environmental pollutants such as PM, PM 2.5 (Outdoor and Indoor), PM 10, CO, CO_2_, CH4 (Methane), NO_2_ (Nitrogen Dioxide), even for extended periods, which can lead to an increase or worsening of depressive symptoms (Szyszkowicz et al., 2016; Kioumourtzoglou et al., 2017; Lin et al., 2017). Air pollution has consistently been associated with increases in pro-inflammatory biomarkers in the blood and systemic oxidative stress, both involved in the pathogenesis of psychiatric disorders such as MDD (Jones et al., 2013; Buoli et al., 2018). Based on current scientific evidence (Ventriglio et al., 2021; Cuijpers et al., 2023), cumulative exposure to pollution can be measured with environmental devices essential for monitoring air quality and pollutant concentrations in the home environment. Also the prolonged exposure to air pollution may adversely affect mental health, underscoring its associations with MDD (Nobile et al., 2023). Comparing short-term and long-term exposure to environmental pollutants with the development of MDD can provide interesting data from a prognostic perspective regarding integrating technology into mental health prevention and treatment processes.

### 1.3 Digital biomarkers and MDD

Although biological biomarkers remain central in determining pathology, significant progress has been made in processing social signals as a diagnostic tool (Scherer et al., 2012; Joshi et al., 2013; Williamson et al., 2013). As a supplement to these methodologies, digital indicators of depression have the potential to contribute valuable insights to clinician evaluations. This is particularly advantageous when these indicators can be effortlessly and inconspicuously gathered outside of traditional clinical settings.

Digital biomarkers, collected through digital devices, offer objective and quantifiable physiological and behavioral data. They enable remote data collection, potentially reducing the burden of in-clinic visits and providing insights into clinically relevant changes (McIntyre et al., 2023). Wearable technologies and smartphone apps contribute to digital phenotyping, allowing for moment-by-moment assessment of individual characteristics in real-world environments. These tools have been investigated for their utility in characterizing and diagnosing MDD, including analyzing patterns of physical activity, voice samples, light exposure, and smartphone usage data (Lee S. et al., 2021; Ahmed A. et al., 2023).

The integration of digital biomarkers for depression holds the promise of improving clinical interventions (Mohr et al., 2017). This improvement can manifest through prompt identification for early intervention, continuous assessment during treatment, and the mitigation of disparities in assessment accessibility (Kumar and Phookun, 2016; Naslund et al., 2017).

Voice analytics has shown promise for detecting symptoms of depression (Soskin et al., 2012; Beurel et al., 2020).

The idea of using vocal acoustic features as potential biomarkers for identifying or diagnosing depression is an emerging area of research within the field of computational psychiatry and affective computing. The premise behind this approach is that changes in vocal characteristics, such as pitch, tone, rhythm, and intensity, may reflect underlying emotional states, including depression (Schwoebel et al., 2021; Sverdlov et al., 2021).

Several studies have explored the relationship between vocal features and depression (Mundt et al., 2012; Huang et al., 2021). These studies typically involve collecting speech samples from individuals diagnosed with depression and comparing their vocal characteristics with those of non-depressed individuals. Some common findings include:

1. **Pitch**: Depressed individuals may exhibit alterations in pitch, such as reduced pitch variability or overall lower pitch compared to non-depressed individuals.
2. **Tone**: Changes in tone, including flatter or more monotone speech, have been observed in individuals with depression.
3. **Speech rate and rhythm**: Depressed individuals may speak at a slower rate or exhibit disrupted speech rhythm compared to non-depressed individuals.
4. **Intensity**: Variations in speech intensity, including reduced loudness or energy in speech, have been linked to depression.
5. **Prosody**: Depressed individuals may demonstrate abnormalities in prosody, which refers to the patterns of stress and intonation in speech.

Researchers have developed computational algorithms to analyze these vocal features automatically, allowing for objective and quantitative assessment of depressive symptoms. Machine learning (ML) techniques, such as support vector machines, neural networks, and Gaussian mixture models, have been employed to classify individuals as depressed or non-depressed based on their vocal characteristics (Zhao et al., 2022).

While the potential of vocal acoustic features as biomarkers for depression is promising, several challenges remain. These include the need for large-scale validation studies, addressing confounding factors such as age, gender, and cultural differences, and ensuring the privacy and ethical use of speech data in clinical settings.

### 1.4 The role of AI in MDD

Integrating computer technologies and AI is a cutting-edge approach in mental health research (Graham et al., 2019), addressing the growing challenges faced by healthcare systems. AI, especially ML, aids in the precise and rapid diagnosis of mental health disorders by analyzing biomarkers related to patients' biological, physiological, and behavioral aspects (Iyortsuun et al., 2023). AI holds promise in understanding the complex interplay of biological, psychological, and social factors in mental health. The use of AI and wearable devices allows for continuous, objective patient monitoring, offering insights into mood changes and treatment effects, thereby enhancing mental health care and research (Nahavandi et al., 2022). Integrating AI algorithms with real-time data from wearables and sensors marks a substantial stride forward, enabling the creation of personalized and responsive mental health systems (Boucher et al., 2021; Omarov et al., 2023). Personal wearable devices like smartwatches and fitness trackers offer real-time data collection for healthcare. They are valuable in monitoring and assessing patients with depression, as they capture data related to physical activity and smartphone usage, among others. Advancements in smartphone-based speech analysis and wearable technologies like smartwatches offer new opportunities for real-life depression screening and the generation of digital biomarkers (Sequeira and Perrotta, 2020; Lee Y. et al., 2021). Research on digital biomarkers addresses four fundamental aspects: forecasting diagnostic status, evaluating symptom severity and progression, discerning treatment response, and monitoring real-world and ecological (Ahmed A. A. M. et al., 2023; Vignapiano et al., 2023). Various wearable technologies have been employed to gather physiological, activity/sleep, or subjective data in order to investigate their correlations with depression.

The next step to apply these findings clinically is to integrate relevant measures into non-invasive and cost-effective tools for use in clinical settings. This integration, including physical, psychopathological, and environmental indicators, could enhance clinical assessments by reducing inter-rater subjectivity relative to diagnosis and symptom evaluation and treatment response (McGuire et al., 2015).

Digital psychiatry integrates various healthcare aspects such as delivery, illness surveillance, disease management, and treatment, with advancements in AI and ML facilitating the translation of new data into clinically relevant digital biomarkers (Orsolini et al., 2024). Wearable devices are expected to play a crucial role in personalized telemedicine, with ongoing research aiming to enhance digital phenotyping precision and efficacy in mental healthcare, ultimately improving the quality of life for individuals with MDD (Courtet et al., 2023).

### 1.5 Aim of the study

This study focuses on evaluating specific digital and environmental biomarkers in adolescents and young adults diagnosed with MDD. The study, conducted through a prospective investigation on the MDD population (i.e., MDD), aims to:

1. Evaluate digital, environmental, and blood biomarkers as predictive indices of recurrence or remission of depressive pathology.
2. Enhance digital technologies useful for predicting the severity of psychopathology and assessing treatment effectiveness.
3. Apply technologies to distinguish individuals with depression and determine a personalized approach.
4. Predict the severity of psychopathology among subjects using biomarkers derived from digital tools.

## 2 Methods

**Figure 1:**
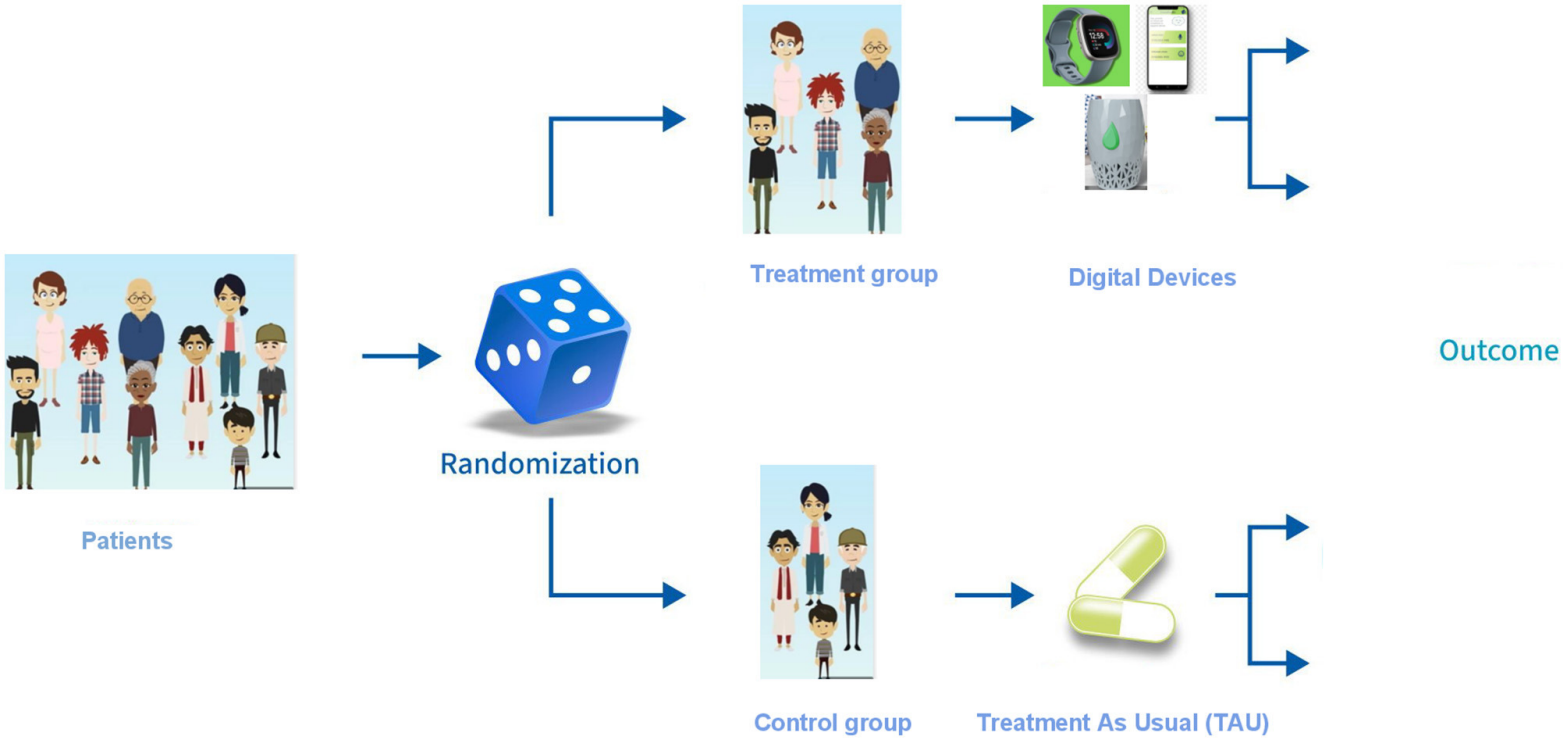
Study design.

**Figure 2:**
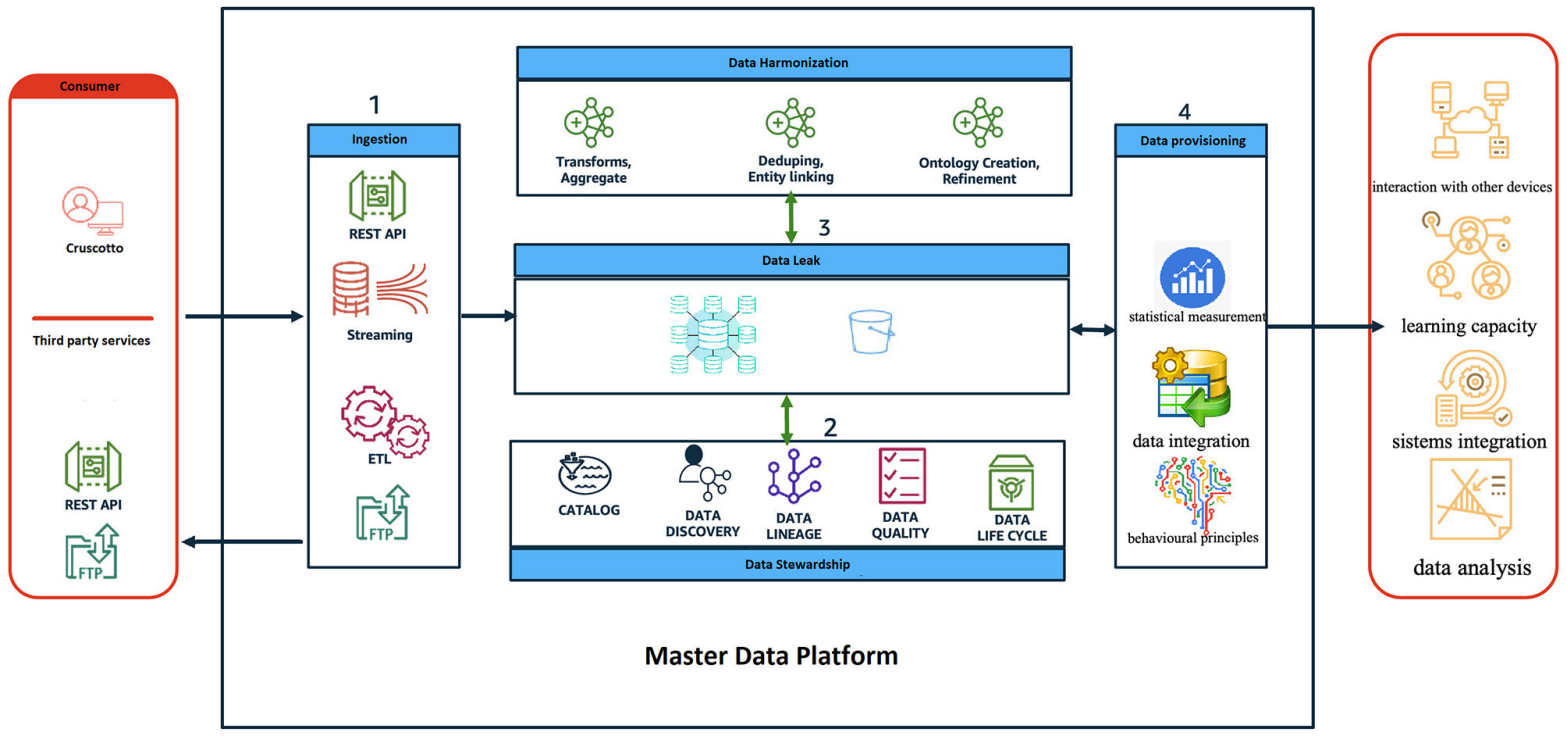
Master data platform.

This project, a prospective, randomized, interventional, and non-pharmacological integrated study (Figure 1), proposes a novel technological platform designed to collect, evaluate, and analyze a comprehensive range of digital and environmental biomarkers in adolescents and young adults diagnosed with MDD, through a prospective 24 months study. The platform adheres to principles of openness, integrability, and scalability to facilitate future advancements and its main components are described in Figure 2.

- **Sensor integration:** The platform utilizes a core hub capable of integrating various sensors and smart devices (wearables, smartphones, “Pothos” air purifier) to collect physiological (e.g., heart rate, oxygen saturation), behavioral (e.g., activity, sleep, voice), and environmental data (e.g., CO_2_, fine dust, environmental brightness). The platform specifically uses Android-based smartwatches and smartphones because this software allows for easier integration with experimental programs like this one.
- **Mobile app:** An individualized mobile application will be developed by IT.Svil. s.r.l. with the purpose of enhancing patient engagement and simplifying communication with healthcare providers. This app will facilitate the collection of self-reported data from patients, thereby streamlining the healthcare process.
- **Data management:** To encourage collaboration among various components, a hub-and-spoke topology will be adopted. This approach ensures efficient interaction between different entities. The utilization of commercial cloud platforms is anticipated to optimize performance, cost-effectiveness, and security. Moreover, in line with privacy by design principles, the platform will prioritize data security through measures such as secure communication channels, VPN, MFA, and data encryption and anonymization techniques.
- **Deep learning analysis:** The platform utilizes advanced DL algorithms to analyze the collected data. These algorithms, based on neural networks, are adept at handling complex and diverse datasets. This is crucial because the platform will gather a wide range of information such as data types (e.g., digital, environmental, and biological) and data formats both discrete and continuous. By analyzing this data, the platform aims to uncover hidden patterns that can help to: improve diagnosis and tailor treatment plans to the individual's specific needs. The focus on DL underlines the platform's aim to become a modern and innovative decision support system that goes beyond simple data analysis.
- **Data standardization and applications:** Data will be structured according to international healthcare standards (e.g., International Patient Summary, IPS) for clear communication and analysis. Additionally, value-added applications for data visualization beyond basic time series dashboards are envisioned. IT.Svil. s.r.l.'s IT technicians, in collaboration with the research team, will undertake the development of the data visualization dashboard. This collaborative effort will ensure comprehensive input from various team members, resulting in an efficient and effective dashboard design.

The experimental sample included 80 subjects aged 14 to 50 diagnosed with MDD (after administration of SCID-5 CV) who have been on stable medication for at least 16 weeks.

To achieve a 95% confidence level with a 5% margin of error in statistical sampling, considering a population proportion of approximately 50% and a known population size constituting 80% of the total, a minimum of 67 patients will be required. This calculation incorporates factors like the Z-score, standard deviation, and a finite population correction for larger populations. The meticulous consideration of these elements ensures the reliability and precision of the sample size, thus enhancing the credibility of the statistical findings in representing the broader population.

Participants will be excluded if they presented: intellectual disability; current or previously diagnosed psychotic disorder; mood disorder with psychotic features; bipolar disorder; alcohol and drug use with psychotropic effects according to DSM-5; pathologies of a chronic inflammatory nature; history of gastrointestinal disorders in the previous week; pregnancy.

The sample will be divided into two age groups (14–17 years and 18–49 years) and distributed in a randomized manner by random allocation in two different groups (group A and group B), each consisting of 40 subjects (10 adolescents and 30 adults).

Based on data from the Italian Statistical Institute (ISTAT) regarding the incidence of depression among italian adolescents, 20 teenager were included in the total sample.

Only the subjects assigned to group B will receive the technological equipment (Pothos system and smartwatch) for the collection of digital biomarkers; group A will be followed according to clinical standards of care. Specialized medical personnel will follow both groups from a pharmacological point of view.

The assessment will include socio-demographic information, clinical history, medication details, and various clinical scales for evaluating depression severity (HAM-D, MADRS, CDI-2) and overall functioning (GAF, C-GAS, CBCL 6–18). Clinical parameters that will be monitored daily include: heart rate, and respiratory rate, oxygen saturation, light intensity, sleep/wake rhythm, and daily step count. The daily mood will be detected by using specific emoticons by the study participants. Additionally, vocal analysis will be conducted weekly using an “*ad hoc*” app to evaluate global articulation rate, fundamental frequency, and energy during specific tasks. Specific characteristics to be analyzed include F0 range (Hz), Energy per second (mV2), Average pause duration (s), Total pause time (s), and Variability of pauses (s).

The study will also include the collection of blood and saliva samples to assess inflammatory biomarkers (IL-1β, IL-2, IL-4, IL-5, IL-6, IL-10, IL-12, and IFNγ) and cortisol levels at the beginning (T0) and the end of the study (T1). Samples of peripheral blood and saliva will be taken in the group of patients with MDD and controls.

Blood withdrawal is necessary to recover the plasma and dose cytokines of pro- and anti-inflammatory cascade; the salivary sample allows the dosage of cortisol instead. Blood (7 ml) will be collected in K_2_-EDTA tubes, and plasma will be obtained through a density gradient centrifugation using SepMate™ Tubes. According to the manufacturer's instruction, blood will be diluted with PBS and then stratified on a gradient density medium (LymphoSep™, Biowest) that will allow the separation of plasma and other components after centrifugation at 1,200 × g for 10 min at a room temperature, brake on. Plasma will be recovered and stored at −80°C until it is used.

Saliva (>1 ml) will be collected in the morning, before brushing teeth and consuming food, through the use of a Salivette^®^ (Sarstedt), a test tube containing a swab to be chewed slowly for 2 min. Salivette^®^ will be centrifuged at 1,500 × g for 10 min at 4°C and then saliva will be recovered and stored at −20°C until the use.

IL-1β, IL-2, IL-4, IL-5, IL-6, IL-10, IL-12, and IFNγ inflammatory biomarkers will be dosed in plasma through the Elisa test using ELISA Pro:Human Kits (Mabtech). IL-1α will be measured by using Interleukin-1α (human) ELISA kit (Cayman); cortisol will be quantified in saliva through a competitive assay (Cortisol ELISA Kit, Cayman). All the Elisa tests will be performed according to the manufacturer's instructions.

The study protocol and informed consent form were approved by the Campania Sud Review Board (application protocol: 48_r.p.s.o.).

## 3 Results

The study's sample will first undergo initial age-based stratification. Subsequently, a more detailed categorization will be conducted based on outcomes observed during a 6-month follow-up period. This classification relies on remission, a crucial parameter indicating intervention effectiveness, determined by a HAM-D score ≤ 7. This stratification acknowledges age's impact on MDD manifestation and allows refined analysis of treatment outcomes in distinct age groups. Remission or clinical improvement presence will be confirmed using data from digital and environmental biomarkers, exploring connections between biomarkers, depression expression, disease course, and early illness signs.

## 4 Conclusions

The study endeavors to assess biomarkers as predictive indicators for the relapse or remission of MDD, with the overarching goals of advancing digital technologies in predicting psychopathological severity, evaluating treatment efficacy, distinguishing individuals with MDD, and forecasting psychopathology severity through digital biomarkers. Simultaneously, this study seeks to validate an AI tool for early MDD diagnosis, implement an AI solution for continuous data processing, and establish an AI infrastructure for healthcare Big Data management. The integration of innovative psychophysical assessment tools in clinical practice is pivotal, aiming to enhance diagnostic efficiency and develop more specific digital devices for comprehensive mental health evaluation.

## Ethics statement

The study protocol and informed consent form have been approved by the Campania Sud Review Board (Application no. 48_r.p.s.o.).

## Author contributions

FM: Conceptualization, Methodology, Supervision, Writing – original draft, Writing – review & editing. AV: Conceptualization, Methodology, Supervision, Writing – original draft, Writing – review & editing. MP: Data curation, Methodology, Writing – original draft, Writing – review & editing. FF: Conceptualization, Data curation, Methodology, Writing – original draft, Writing – review & editing. CP: Conceptualization, Funding acquisition, Project administration, Resources, Writing – original draft, Writing – review & editing. AM: Conceptualization, Data curation, Methodology, Writing – original draft, Writing – review & editing. SL: Conceptualization, Data curation, Methodology, Writing – original draft, Writing – review & editing. CV: Writing – original draft, Writing – review & editing. CM: Funding acquisition, Project administration, Writing – original draft, Writing – review & editing. VP: Resources, Writing – original draft, Writing – review & editing. DI: Writing – original draft, Writing – review & editing. AA: Writing – original draft, Writing – review & editing. RC: Writing – original draft, Writing – review & editing. AD'A: Writing – original draft, Writing – review & editing. MF: Methodology, Supervision, Writing – original draft, Writing – review & editing. MS: Writing – review & editing, Supervision, Writing – original draft. GC: Funding acquisition, Resources, Supervision, Writing – original draft, Writing – review & editing, Validation. AF: Funding acquisition, Resources, Supervision, Writing – original draft, Writing – review & editing.
